# Some Molecular and Cellular Stress Mechanisms Associated with Neurodegenerative Diseases and Atherosclerosis

**DOI:** 10.3390/ijms22020699

**Published:** 2021-01-12

**Authors:** Margarita A. Sazonova, Vasily V. Sinyov, Anastasia I. Ryzhkova, Marina D. Sazonova, Tatiana V. Kirichenko, Victoria A. Khotina, Zukhra B. Khasanova, Natalya A. Doroschuk, Vasily P. Karagodin, Alexander N. Orekhov, Igor A. Sobenin

**Affiliations:** 1 Laboratory of Angiopathology, Institute of General Pathology and Pathophysiology, Russian Academy of Medical Sciences, 125315 Moscow, Russia; centaureaceanus@mail.ru (V.V.S.); ryzhkovaai@gmail.com (A.I.R.); marinasazon1990@gmail.com (M.D.S.); t-gorchakova@mail.ru (T.V.K.); nafany905@gmail.com (V.A.K.); vpka@mail.ru (V.P.K.); a.h.opexob@gmail.com (A.N.O.); igor.sobenin@gmail.com (I.A.S.); 2 Laboratory of Medical Genetics, National Medical Research Center of Cardiology, 121552 Moscow, Russia; zukhra@yandex.ru (Z.B.K.); natador28@mail.ru (N.A.D.); 3 Laboratory of Cellular and Molecular Pathology of Cardiovascular System, Research Institute of Human Morphology, 117418 Moscow, Russia; 4 Department of Commodity Science and Expertise, Plekhanov Russian University of Economics, 125993 Moscow, Russia; 5 Institute for Atherosclerosis Research, Skolkovo Innovative Centre, 143024 Moscow, Russia

**Keywords:** chronic stress, oxidative stress, genotoxic stress, cellular stress, mutation, gene, mitochondrial genome, nuclear genome

## Abstract

Chronic stress is a combination of nonspecific adaptive reactions of the body to the influence of various adverse stress factors which disrupt its homeostasis, and it is also a corresponding state of the organism’s nervous system (or the body in general). We hypothesized that chronic stress may be one of the causes occurence of several molecular and cellular types of stress. We analyzed literary sources and considered most of these types of stress in our review article. We examined genes and mutations of nuclear and mitochondrial genomes and also molecular variants which lead to various types of stress. The end result of chronic stress can be metabolic disturbance in humans and animals, leading to accumulation of reactive oxygen species (ROS), oxidative stress, energy deficiency in cells (due to a decrease in ATP synthesis) and mitochondrial dysfunction. These changes can last for the lifetime and lead to severe pathologies, including neurodegenerative diseases and atherosclerosis. The analysis of literature allowed us to conclude that under the influence of chronic stress, metabolism in the human body can be disrupted, mutations of the mitochondrial and nuclear genome and dysfunction of cells and their compartments can occur. As a result of these processes, oxidative, genotoxic, and cellular stress can occur. Therefore, chronic stress can be one of the causes forthe occurrence and development of neurodegenerative diseases and atherosclerosis. In particular, chronic stress can play a large role in the occurrence and development of oxidative, genotoxic, and cellular types of stress.

## 1. Introduction

Chronic stress is a combination of nonspecific adaptive reactions of the body to the effects of various adverse stress factors that disrupt its homeostasis, as well as the corresponding state of the nervous system of the body (or the body as a whole) [1,2]. Under chronic stress, internal changes in the systems of a human body occur in response to a strong or prolonged exposure to the environmental stressor [3,4,5,6,7]. Chronic stress harms physical and mental health of people of all genders, races, and ages [8,9]. Stress happens if mental, emotional, and/or physical demands rise higher than regulatory possibilities of a body [8,10] and the influence on an organism can vary depending on the frequency, magnitude, and length of the stress. While moderate levels of stress can be adaptive, stress persisting for long periods can have negative consequences on the well-being of an organism [8,11]. Several preclinical and clinical researches have found that chronic stress produces alterations in gray and white matter of the brain, and affect healthy neural communication through changes in brain circuits [8,12]. Moreover, chronic stress is linked with several psychiatric illnesses, for example, depression, anxiety, post-traumatic stress disorder (PTSD), substance use disorder (SUD), and personality disorders [8,13].

Chronic stress refers to the non-specific systemic reaction which happens if the body is stimulated by different internal or external negative factors during a long period of time [14,15,16]. The physiological response to chronic stress exposure has long been recognized as a potent modulator in the occurrence of atherosclerosis. Moreover, studies have confirmed that there is a correlation between atherosclerosis and cardiovascular events. Chronic stress is pervasive during negative life events and can cause the formation of atherosclerotic plaques [14,15,16]. The results of a number of epidemiological researches have shown that chronic stress is an independent risk factor for the development of vascular diseases and for higher morbidity and mortality in patients with pre-existing coronary artery disease. One possible mechanism for this process is that chronic stress causes endothelial injury, directly activating macrophages, promoting foam cell formation and triggering the formation of atherosclerotic plaques [14,15,16]. This mechanism involves numerous variables, which include inflammation, signal pathways, lipid metabolism and endothelial function. The mechanisms of chronic stress in atherosclerosis should be further investigated to give a theoretical basis for efforts to liquidate the negative influence of chronic stress on the cardiocerebral vascular system [14,15,16].

In response to chronic stress, changes in an individual’s metabolism can occur (increased levels of lipids and glucose in blood). This prepares the organism to enhanced muscular activity, which was necessary in the struggle for the existence of the individual in ancient times. Although at present, there is almost no need for a person to use muscular activity for their own survival, the pathophysiological mechanisms of chronic stress have not changed much. Since the psychological conflicts of modern people do not involve physical manifestations, this contributes to the excessive accumulation of glucose, lipids, and other substances produced by the organism as a result of stress, which triggers atherogenesis [17,18]. As a result, athletes or workers who perform physical labour a lot are much less likely to have atherosclerotic lesions than people engaged in mental work and leading a sedentary lifestyle.

According to a study conducted by Finnish scientists, in individuals with a high level of flow-mediated dilatation, a slower recovery of the pre-ejection period is a prognostic factor in the thickening of the intimal-medial layer. Scientists suppose that endothelial dysfunction is one of pathophysiological mechanisms which associate with atherosclerosis the slow recovery of cardiac activity after completing a task caused by chronic stress in the conducted experiment [19].

Hemodynamic responses to neuropsychic stress are also a risk factor for atherosclerosis. Scientists from the United States found that the increase in systolic blood pressure triggered by psychological problems can be a prognostic factor for atherosclerosis in men older than 40 years. In the investigated sample, this fact did not depend on other risk factors [20].

Researchers from Boston suggested that atherosclerotic lesions can affect the vasomotor reactions of coronary arteries under neuropsychic stress. The data obtained by scientists confirmed that vasomotor reactions in atherosclerosis differ from normal [21].

Therefore, chronic stress can lead to the occurrence and development of neuropsychic diseases and atherosclerosis. We suppose that a key role in their pathogenesis can play molecular and cell types of stress. At present, there are several types of classification of these types of stress. In our review article we would like to present a classification of molecular and cell types of stress from the point of view of medical molecular geneticists.

## 2. Molecular Types of Stress

Oxidative stress and genotoxic stress belong to molecular types of stress. Besides, DNA methylation caused by stress, can be included into this group, too.

### 2.1. Oxidative Stress

Redox reactions underlie the molecular biological processes in humans. In particular, they participate in metabolic processes which are linked with protein phosphorylation, induction of the Ca^2+^-signal, modulation of transcription factors, and hydrolysis of phospholipids [22,23,24,25].

Oxidative stress is a process of damaging of various organs and tissues by reactive oxygen species at the cellular level. It arises as a result of an imbalance in the functional activity of the prooxidant and antioxidant systems that support or inhibit oxidation (respectively). This process can be a result of both the lack of antioxidant protection caused by a disruption in the production of endogenous antioxidants and a superfluous excess of free radicals formed [26,27,28,29].

Since most free radicals are cytotoxic, they are included in pathogenetic mechanisms. Free radicals can be by-products of metabolic processes. Among them, the “reactive oxygen species” (ROS) play a large role (Table 1) [30,31,32,33,34].

In any stressful reactions accompanied by oxidative stress, ROS are included in the transmission of information from primary mediators: hormones, cytokines, neurotransmitters through the cell membrane to trigger reactions of adaptation the organism to extreme conditions. Pathological processes in the human body can occur due to sharp fluctuations in the concentration of ROS in cells and tissues. For example, activation of apoptosis and proliferation genes may begin, and the level of cytokine expression may increase. In addition, somatic DNA mutations and cytotoxicity may occur [35,36,37,38,39].

The DNA molecule may be damaged by hydroxyl radical and superoxide anion. Hydroxyl radical may cause oxidation of purine and pyrimidine bases, as well as on residues of ribose and deoxyribose. Superoxide anion interacts with guanine bases, as a result of which their various oxidized derivatives are formed, including the final oxidation product of guanine bases, 7,8-dihydro-8-hydroxyguanosine. Radicals formed during lipid peroxidation also damage DNA molecules. Mitochondrial DNA is subjected to the oxidative effect of ROS even more than the nuclear one, since it is in close proximity to ROS sources and is not protected by histones. During the interaction of hydrogen peroxide, formed in the respiratory chain, with ions of Fe^2+^ and Cu^2+^, which are present in mitochondrial membranes, forms hydroxyl radical, which damages mtDNA. Mitochondrial genome can also be damaged by the action of hydrogen peroxide formed in the monoamine oxidase reaction. Damage to mtDNA leads to dysfunction of respiratory chain in mitochondria, resulting in increased wastage of superoxide anion. Damage to mtDNA also occurs as a result of the action of endonucleases, which are activated during the increase in concentration of intracellular Ca^2+^, observed during oxidative stress. As a result of respiratory chain dysfunction in mitochondria, ATP synthesis decreases. In mitochondria and cells of the organism, energy deficiency begins. This leads to oxidative stress. Ultimately, these processes can enhance the production of oxidants. Moreover, during stress, the amount of ROS significantly exceeds the activity of endogenous antioxidant systems which ensure their elimination [30,31].

Oxidative stress leads to damaging of vascular endothelium, arterial hypertension and atherosclerosis. At the molecular level, it leads to damage to nucleic acids, proteins, lipids and the emergence of cell mutations. For example, an increase in the level of hydrogen peroxide in the plasma of patients with arterial hypertension was found. In addition, this indicator was greater in such patients with arterial hypertension, in whom this disease was observed in the family [32].

Endothelial dysfunction and smooth muscle hypertrophy of vascular cells occurring in arterial hypertension and atherosclerosis result from the excessive formation of O_2_^−^. This leads to oxidative inactivation of NO and a decrease in its bioavailability. This ROS is formed using enzyme NO synthase, which is found in macrophages. Its expression occurs with the development of inflammation, in response to bacterial endotoxins and cytokines [33,34,35,36,37].

It should be noted that O_2_^−^ can cause unlimited proliferation of endothelial cells and lymphocytes. At the same time, H_2_O_2_ and NO^−^ inhibit this proliferation. OH^−^ is characterized by the destruction of any CH bonds. RO^−^ and OH^−^ have a carcinogenic effect. O_2_^1^ and OH^−^ are mutagens. To sum it up, an excess of any ROS causes cytotoxicity [38,39,40,41].

#### The Link of Oxidative Stress with Neurodegenerative Diseases and Mitochondrial Genome Mutations

It is believed that mitochondrial dysfunction, which disrupts the structure of mitochondria, is a key element of oxidative stress in the cell. It leads to an imbalance between the production of free radicals and the protective capabilities of the organism [41,42,43,44,45]. It should be noted that oxidative stress can lead to neurodegenerative and vascular diseases, and also to metabolic diseases and cancer [42,43,44,45,46,47].

Parkinson’s disease (PD) is one of the most common neurodegenerative diseases and is clinically characterized by progressive hypokinesia, muscle rigidity and resting tremor [48,49]. The death of dopaminergic neurons of the substantia nigra lies at the core of PD. Meanwhile, in PD Lewy bodies are found in the surviving cells, and the amount of dopamine in the striatum is reduced [48,49,50]. During the study of autopsy material, a defect of complex I of the electron transport respiratory chain of mitochondria of the substantia nigra cells was found, leading to a 30–40% decrease in their activity [51]. This mechanism is determined by the reduced production of subunits of this complex, the destruction of its structure and direct oxidative damage to the cells themselves [52,53]. Even stronger evidence of the link of PD with oxidative stress was obtained by histochemical analysis of substantia nigra cells, in which mtDNA mutations, glycation and nitration of cellular proteins were detected. This leads to the formation of the most significant lipid oxidation product: 4-hydroxy-2 nonenal, which triggers the process of cell death. A change in the conformational structure of the α-synuclein protein is integrated into this mechanism. The aggregates of α-synuclein protein have a toxic effect and are a basis for the formation of Lewy bodies [54]. Chen, S.H. et al. found that changes in oxidative stress and the number of mitochondrial DNA copies y numbers in patients with Parkinson’s disease (PD), especially in those who underwent therapy with the use of dopamine for a long time undergoing long-term dopamine therapy. In particular, this research group evaluated the number of mitochondrial copies numbers, thiobarbituricacid reactive substances (TBARS), and thiols inpatients with Parkinson’s disease andin conditionally healthy people. The total prescribed dopamine dose was calculated for each PD patient. Each patient with Parkinson’s disease was prescribed dopamine, and the dose was different and depended on many factors. Patients with Parkinson’s disease had a decreased low number of mitochondrial copies and antioxidant thiols level, but an increased oxidative TBARS level. The number of mitochondria and thiols decreased with age in all PD subgroups, but they showed high TBARS levels compared to conditionally halthy people. It was found in further investigations that there was an association betweendecreased serum TBARS and dopamine administration. It was detected that a decrease in TBARS diminished the negative effect of pathogenetic and age-related decrease in mitochondrial copy number in patients with Parkinson’s disease. The authors of the study suppose that moderate dopamine dose therapy benefits patients with Parkinson’s disease through attenuation of oxidative stress and manipulation with the number mitochondrial copies [55].

A recent article was published by Nido, G.S. et al., which reports on deletion analysis in individual neurons. It was noted that in Parkinson’s disease at least one small deletion in mtDNA was detected in each of the affected neurons. The most common deletion in the mitochondrial genome for Parkinson’s disease was 4977 bp “common” deletion. The authors suppose that precisely mtDNA deletions cause mitochondrial dysfunction leading to Parkinson’s disease [56,57].

It should be noted that Parkinson’s disease is also associated with mutations in the genes of nuclear genome (SNCA (PARK1), PARK2, PINK1 (PARK6), DJ-1 (PARK7), LRRK2 (PARK8), ATP13A2 (PARK9), PLA2G6 (PARK14), FBXO7 (PARK15), VPS35 (PARK17), EIF4G1 (PARK18), COQ2, DNAJC6, ATP6AP2, SYNJ1, DNAJC13), which lead to oxidative stress, apoptosis or stress in the endoplasmic reticulum [58,59,60,61,62].

Another neurodegenerative disease associated with oxidative stress and mitochondrial dysfunction is Alzheimer’s disease. Alzheimer’s disease (AD) is predominantly a disease of late age, characterized by progressive cognitive decline and leading to the loss of independence in everyday life. The disease is based on irreversible cell death, especially in the cerebral cortex and hippocampus. The main pathomorphological correlates of AD are extracellular aggregates of beta-amyloid and also intracellular neurofibrillary tangles, formed from hyperphosphorylated tau protein. In AD, oxidative stress is one of the key elements in pathogenesis. It is oxidative stress which can lead to disruption of the metabolism of the protein, a precursor of amyloid [63,64]. Tau hyperphosphorylation is the second key pathogenetic mechanism of Alzheimer’s disease, not only due to the fact that it violates the structure of microtubules and axonal transport, but also due to the fact that the absence of a normal tau protein itself leads to the destruction of microtubules [65,66]. The results of recent studies suggest that neurofibrillary glomerular accumulation is also associated with oxidative stress [67,68].

Neuritic plaque, one of the major characteristics of AD neuropathology, mainly consists of amyloid β (Aβ) protein. Aβ is derived from amyloid precursor protein (APP) by sequential cleavages of β- and γ-secretase. Although APP upregulation can promote AD pathogenesis by facilitating Aβ production, growing evidence indicates that aberrant post-translational modifications and trafficking of APP play a pivotal role in AD pathogenesis by dysregulating APP processing and Aβ generation [69].

Recently it was described a mitochondrial matrix peptidase, called PITRM1 (hPreP) that works as scavenger preventing the accumulation of Aβ inside the mitochondria. Alikhani et al. performed a post-mortem study on AD brain samples, showing a significant reduction in proteolytic activity of PITRM1 in the mitochondrial matrix extracted from temporal lobes of AD patients compared with age-matched non-AD individuals. Lower enzymatic activity was not due to a reduced protein expression but to a functional alteration of the enzyme, and possibly through post-translational modifications such as protein oxidation [70,71,72,73,74]. This enzyme in fact, resulted particularly sensitive to oxidizing conditions, because of the presence of two cysteines linked by a disulfide bridge which locks the enzyme in a closed conformation thus hindering substrate binding. Mutations in Pitrm1 are linked with a neurodegenerative disease characterized by ataxia and cognitive decline [75]. Further it was proved that PITRM1 LOF induces proteotoxic stress and Alzheimer’s disease-like pathology in human cerebral organoids [76].

It should be noted that Alzheimer’s disease was found to be associated with 4977 bp “common” deletion, as well as Parkinson’s disease was [77,78,79]. Alzheimer’s disease is also associated with mutations in nuclear DNA genes, such as *APP*, *PSEN1*, *PSEN2,* and *APOE* [80,81,82,83,84].

However, a number of research groups give data on the fact that some neurodegenerative diseases (in particular, major depressive disorder), were not associated with a range of genes-candidates during whole genome sequencing (NGS) [85]. At the same time, during the analysis of separate polymorphisms, this link was earlier detected. For example, such association was detected for 5-HTTLPR variable number tandem repeat (VNTR) polymorphism in the promoter region of the serotonin transporter gene *SLC6A4* [86]. Besides, for one of alleles of this polymorphism, was found an association between stress and anxiety symptoms, which is moderated by 5-HTTLPR [87]. At present, there are several hypotheses and theories, which explain the cause why the results of analysis of separate polymorphisms and NGS are different [85,88,89]. Our research group faced a similar problem while comparing the results of analysis of separate mtDNA mutations by the method of pyrosequencing and the analysis of mutations of whole mitochondrial genome by the method of NGS which were associated with atherosclerosis. In our review article “Mitochondrial genome sequencing in atherosclerosis: what’s next?” we give examples of results of analyses of mutations by research groups from different countries of the world [88]. These investigations were carried out using different methods, for example, the analysis of separate mutations and polymorphisms using PCR, Sanger sequencing and NGS. Meanwhile, these results could be fully or partially do not match [85,88,89]. For example, in our study, some mtDNA mutations were detected during the analysis of separate mutations, but they were not detected using the method of NGS. Meanwhile, our sample for analysis of separate mutations of mtDNA by the method of pyrosequencing was more than 700 individuals, and our sample for analysis of whole mitochondrial genome by the method of NGS, was 115. Perhaps, the difference in the list of the detected mutations was connected with the high cost of NGS and the absence of possibility to sequence the large number of samples, which can be compared to the large number of samples, taken for the analysis of single nucleotide polymorphism or mutation [85,88,89]. We suppose that the comparison of data, obtained on different samples of patients, may be correct, if it was carried out using the same method.

### 2.2. Genotoxic Stress

In genotoxic stress, genome damage occurs under the influence of genotoxins. Genotoxic types of nuclear DNA damage include alkylation, hydrolysis and oxidation. In this case, there occur sister chromatid exchanges, large and small DNA deletions, reading frame shift mutations, chromosomal aberrations, point mutations, cross-linking with other bases and proteins, and DNA strand breaks [90,91].

#### 2.2.1. Replicative Stress

One of the genotoxic stress forms is replicative stress. During this form of stress, a stop of the replicative fork happens because of the occurrence of single-stranded DNA breaks. During replication, erroneous inclusion of non-complementary nucleotides (mismatches) can take place, leading to the occurrence of point mutations. A mutation with a shift in the reading frame may appear or a number of short tandem repeats may increase. For example, an increase in the number of CAG codons in gene *IT-15* leads to the occurrence of such a nervous system disease as Huntington disease [92,93].

Reactive oxygen species lead to the formation of modified nitrogenous bases. In this case, mutations occur with the replacement of bases: transitions or transversions. If the oxidized base is localized in the promoter region of the gene, transcription is impaired. In case of removing damaged bases with DNA glycosylases, AP sites (abasic site) occur. These sites represent the deoxyribose residues in a DNA molecule lacking a nitrogenous base (“AP”–“apurinic” or “apyrimidinic”). In the absence of further repair, AP sites lead to the replacement of damaged nitrogenous bases with thymine or single and double stranded DNA breaks. By some modified bases, DNA polymerases are blocked [94,95,96]. This causes the replication fork to stop. After this, specific DNA polymerases with reduced complementarity requirements may activate. Driven by these polymerases, replication is renewed, but mutations occur in the resulting daughter chain. This process happens because of the fact that purines and pyrimidines are much more sensitive to modification as mononucleosides and nucleotides than as part of DNA, where they are protected by chromatin packaging. Although DNA and RNA polymerases recognize damaged and modified bases, this recognition is not absolute and they can incorporate damaged nucleotides into DNA [97,98,99].

The link of stress with the number of the mitochondrial genome copies was found during the study of posttraumatic stress disorder (PTSD). In particular, Bersani, F.S. et al. found that mitochondrial DNA copy number is reduced in male combat veterans with PTSD compared to those without PTSD [97]. Kelly J. Brunst et al. elucidated that the number of the mitochondrial genome copies in the placenta during maternal lifetime stress and PTSD symptom in white individuals is reduced more significantly than in representatives of other races [98]. However, scientists need further research to achieve statistically significant results.

#### 2.2.2. Mutation Stress. Genetic Predisposition to Genotoxic Stress

One of the specific forms of replicative stress is mutational stress. Some mutations and polymorphisms of genes which control the functioning of the nervous and immune systems make post-traumatic stress disorder more likely after traumatic situations.

It has been shown that the genes of the dopamine and serotonin receptors controlling the transmission of a nerve impulse, affect the extent of being worried by a traumatic situation, and how well the person recovers after that. In particular, the 7-repeat allele (7R) of the dopamine receptor gene (*DRD4*) is an early marker of future psychological disorder in infants who have experienced a short-term separation from their mother [99,100,101]. It is necessary to note that allele 7R has been the most intensively studied in recent years. It is sometimes called a “gene of adventurism” because its carriers have sufficiently clear, statistically significant behavioral differences from those with other variants of gene *DRD4*. In carriers of allele 7R, on average, a desire to search for novelty-seeking behavior is stronger expressed than in other people. Among the carriers of allele 7R, there are more people with attention deficit hyperactivity disorder [102].

At the same time, young people, having an allele of gene MAOA (monoamine oxidase A) with low-MAOA-activity are predisposed to become antisocial when they experience a variety of adverse experiences, most notably maltreatment in childhood [103]. It should be noted that monoamine oxidases A and B carry out the catabolism of monoamines (hormones, neurotransmitters, exogenous monoamines) through their oxidative deamination. These enzymes regulate the concentration of the above substances in the cell and the organism as a whole. The substrates for MAOA are adrenaline, noradrenaline, serotonin, histamine, and also many phenylethylamine and tryptamine surfactants [104]. MAOB (monoamine oxidase B) substrates are phenylethylamine and dopamine. Both types of monoamine oxidases are located in the outer membrane of mitochondria. Since gene MAOA is on the X chromosome, in women, mutations of this gene may be in a heterozygous state. They are compensated by the normal gene on the second X chromosome. At the same time, in men such compensation does not happen. That is why, gene MAOA is often called a “gene of aggressive behavior” [105].

A group of researchers Igata, N. et al., Caspi, A. et al., Zammit, S. et al., Karg, K. et al. found that a short ‘S’ allele of the 5-hydroxytryptamine-linked polymorphic region polymorphism (5-HTTLPR) is associated with increased depression in a high-stress context [86,87,106,107,108,109]. Insertion deletion polymorphism (5-HTTLPR) is located in the promoter part of the serotonin transporter gene (*SLC6A4*). A possible cause of depression in a high-stress context may be the association of allele “S” with a deletion [86,87,110,111]. Often this occurs in patients in response to adverse environmental influences in childhood [106,111,112]. The search for mechanisms mediating the effect of polymorphism 5-HTTLPR on the development of anxiety and depression led to the detection in allele “S” carriers of enhanced processing of emotionally negative information, including expression of fear and anger on the face. Therefore, the presence ofallele “S” in the genotype turned out to be associated with increased activation of the tonsil of the brain during the perception of frightened and angry faces and a more pronounced negative shift (i.e., a tendency to involuntarily direct attention towards emotionally negative stimuli) compared with homozygous carriage of a long allele (“L”) [106,111,112].

The features of the immune system work are very important in the reaction of the organism to stress factors. People with a genetically determined stronger inflammatory response at high levels of stress are more susceptible to diseases with an inflammatory component (for example, cardiovascular diseases, autoimmune diseases, mental disorders). For example, Seeger et al. evaluated whether the season of the year in which a child was born interacted with the dopamine *DRD4* polymorphism in predicting the hyperkinetic conduct disorder (ADHD). When comparing patients with controls, children with one copy of the *DRD4* 7-repeat allele born in autumn and winter (that is, long photoperiod during pregnancy) had a 5.4-fold decreased relative risk for hyperkinetic conduct disorder, whereas children with the same genotype born in spring and summer (that is, short photoperiod) had a 2.8-fold increased relative risk for hyperkinetic conduct disorder [113,114].

Slominsky, P.A. et al. analyzed the expression of genes related to lysosomal autophagy: *HSPA8*, *LAMP2*, *TFAM*, *SLC18*a*2*, and *VPS35* in the brain tissues of mice with the earliest stage of MPTP-induced PD. Decrease in *HSPA8* and *LAMP2* mRNA levels suggests that dysfunction of lysosomal autophagy may be involved in the earliest stages of PD pathogenesis. A decrease in the rate of lysosomal autophagy may affect the accumulation of damaged proteins and the formation of protein inclusions in PD. Genes related to the lysosome function may be involved in development of both lysosomal storage disorders (LSD) and Parkinson’s disease (PD) at the earliest stages of these pathologies [115,116,117].

### 2.3. DNA Methylation Caused by Stress

Resistance to stress is not only affected by heredity but also by the characteristics of genes which form after birth. After birth, there is a fine adjustment of the work of the genetic apparatus to environmental conditions. Stress conditions also cause this adjustment. As shown in animal models, when stressful situations occur in mousekins and young rats, their genes change their level of activity [118,119]. Subsequently, such a level of activity of these genes can persist until the end of life. For example, separation from the mother is a great stress for young rats. And if the young rat is taken away from the mother for several hours and then returned, the cortisol level and the organizm’s response to stress change, and these changes last for life. In this case, methyl groups join the genes involved in the stress response. The activity of methylated genes may decline for life [118,119]. A study of the molecular mechanisms of the response to stress has been conducted in humans. A group of researches Champagne, D.L. et al. studied children who grew up in a single-parent family, in which the parents divorced or one of the parents died. It turned out that their level of methylation of the cortisol receptor gene was higher than in children who grew up in an intact family. I.e., the intensity of the gene expression levels in these children is reduced. This can lead to the fact that carriers of the methylated gene will react more sharply to a stressful situation [120]. Tyrka, A.R. et al. found that disruption or lack of adequate nurturing, as measured by parental loss, childhood maltreatment, and parental care, was associated with increased *NR3C1* promoter methylation. In addition, *NR3C1* promoter methylation was linked to attenuated cortisol responses to the Dex/CRH test [121].

## 3. Cellular Types of Stress

Several types of stress belong to a group of cellular stresses: the very cellular stress, endoplasmic reticulum stress, and osmotic stress.

### 3.1. Cellular Stress

The reaction of the cell to various types of stress determines its future fate. During cellular stress, the permeability of membranes increases, the membrane potential of cells is depolarized. CO_2_ penetrates into the cytoplasm from cell walls and intracellular compartments. The pH of the cytoplasm decreases. Microfilament assembly is activated. The consequence of this is an increase in the viscosity and light scattering of the cytoplasm. Oxygen and ATP absorption are increasing. The speed of hydrolytic processes is increasing. Stress-induced proteins are activated. Their synthesis begins. The activity of the H+ pomp in the plasmalemma is enhanced. The synthesis of ethylene is increasing. Suppression of cell division and growth occurs. These cell reactions are aimed at protecting intracellular structures and eliminating adverse changes in the cells [122,123,124].

Under the influence of stress factors, apoptosis, autophagy, necrosis and ferroptosis of cells can occur. These stress reactions are characteristic of neurodegenerative diseases [125,126,127]. A key factor in neurodegenerative diseases is the death of a neuron, which can be of three types: programmed cell death (apoptosis), pathological cell death (necrosis) programmed oxidative necrotic cell death (ferroptosis). Moreover, apoptosis, ferroptosis and necrosis of the neuron are morphologically different. In particular, a characteristic feature of ferroptosis is ferrum dependent peroxidation of lipids. For ferroptosis the following morphological changes are characteristic: a decrease in number or the disappearing of cristae in mitochondria, small size of mitochondria with dense inner membranes, ruptures of external membrane of mitochondria [127,128,129,130]. The primary reaction of the neuron to apoptotic effects, apparently, is realized by proto-oncogenes. The activation of these genes is considered as one of the main components of the neuronal response to damage preserved in evolution. Noteworthy is the expression of gene *JUN* in the central nervous system. The product of this gene is regulatory protein c-Jun. It is connected with transcription factors which realize a cellular response to damage through activation or repression of genes. Protein C-Jun is involved in the regulation of the cell cycle, differentiation, organogenesis, tumor transformation and apoptosis. It was found that the activation of the *JUN* proto-oncogen with increased expression of its product, protein c-Jun, occurs in neurodegenerative diseases (amyotrophic lateral sclerosis, Alzheimer’s disease). Some scientists see c-Jun as an early marker of signaling activation in apoptosis [131]. In the effector phase of apoptosis in human neurons, a key role is played by caspases, for example, interleukin-1p-converting protease (ICE) or caspase-1 [132]. Meanwhile, apoptosis is mainly regulated by proteins of the Bcl-2,family, and besides two classes of these proteins are distinguished: inhibiting apoptosis (bcl-w, bcl-2, brag-1, bfl-1, a-1, mcl-1) and inducing this process (bax, bcl-xs, bak, bid, bad, hrk, bik). The ratio of inhibitory and inducing proteins determines the ability of a neuron to respond to apoptosis signals [133]. Groups of scientists Galluzzi, L. at al. and Jiang, L. et al. showed that ferrroptosis is one of mechanisms by which protein-suppressor of tumors p53 maintains homeostasis of the organism during stress [129,130].

In Alzheimer’s disease and amyotrophic lateral sclerosis, an increased expression of gene Bax was detected. It was found that gene of low affinity receptor to nerve growth factor (*pTSNGFR*) induced apoptosis only in the nervous system [134]. Genes involved in the mechanisms of apoptosis also play a role in the occurrence of neuron necrosis. For example, gene *BCL-2* inhibits necrosis [135]. Gene *BCL-XL* inhibits not only apoptosis, but also necrotic neuronal death during hypoxia. ICE protease inhibitors have the same properties. This suggests the presence of common molecular-cellular mechanisms of apoptosis and necrosis [136].

### 3.2. Endoplasmic Reticulum Stress

**Figure 1:**
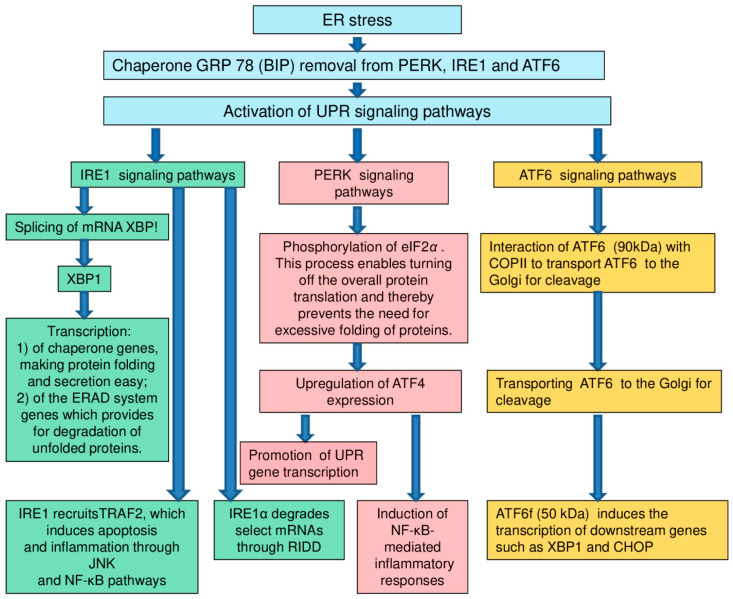
Signaling pathways [128]. Remark: UPR: unfolded protein response; ER: endoplasmic reticulum; IRE1: inositol-requiring enzyme 1; PERK: protein kinase RNA- (PKR-) like kinase; ATF6: activating transcription factor 6; XBP1: X-box-binding protein-1; TRAF2: tumour necrosis factor receptor- (TNFR-) associated factor-2; JNK: Jun-N-terminal kinase; NF-κB: nuclear factor κB; RIDD: regulated IRE1-dependent decay; eIF2α: eukaryotic translation initiation factor 2α; ATF4: activating transcription factor 4; COPII: coat protein II; CHOP: CCAAT/enhancer-binding protein-homologous protein.

Endoplasmic reticulum (ER) is a large membrane organelle which plays an important role in the life-support of a eukaryotic cell. It is involved in the synthesis and modification of proteins, buffering calcium, the synthesis of steroids and the construction of intracellular membranes. In addition, ER takes part in many signaling pathways which regulate gene expression and apoptosis (Figure 1) [137,138,139,140,141].

In many types of cells (primarily those specializing in protein secretion), a significant part of intracellular membranes belongs to ER. The ER membrane is integral with the membrane of the cell nucleus. The ER cavity opens directly into the perinuclear space, which favors the contact of the ER signaling apparatus with genetic material [137,138,139,140,141].

It is ER which provides the synthesis and maturation of proteins intended for secretion or exposure on the surface of the cell membrane. The maturation of any protein molecule implies its “folding”, spontaneous acquisition of the only correct three-dimensional structure (conformation). Violation of the maturation of protein molecules and the accumulation of improperly folded protein chains is the pathophysiological core of “ER stress”. The situation in which the intermediate form of the protein is so unsuccessful that it leads to an unexpected interaction with cellular components is called misfolding or “folding error”. Misfolding poses a real threat to the life of the cell and even the multicellular organism as a whole, laying a foundation for states such as Alzheimer’s disease [142,143,144], autosomal retinitis pigmentosa [145,146,147], α1-antitrypsin deficiency [148,149,150,151], and oncological diseases [152,153,154,155].

The formation of disulfide “bridges” between the side chains of cysteine residues is essential for stabilizing the protein molecule. The formation of S–S bridges in the protein chain sharply limits the number of possible variants of 3D organization, effectively reducing the time for the molecule to search for the correct conformation [156,157,158]. Similar to the process of oxidative phosphorylation in mitochondria, the construction of disulfide bonds using the enzyme Ero1-L inextricably happens with the formation of hydrogen peroxide (H_2_O_2_). This determines a high concentration of H_2_O_2_ in the lumen of the ER and associates the maturation of the secreted protein with the generation of reactive oxygen species which potentially threaten cell survival [159,160,161,162].

More and more data indicate the involvement of ER stress in a wide variety of pathophysiological processes. For example, mutant variant of presenilin-1 protein is involved in stress mechanisms of the endoplasmic reticulum. This protein is a part of the development of Alzheimer’s disease. It inhibits the function of PERK (a protein from the eIF2alpha kinase family, activated in response to various cellular stresses), IRE1α (α-isoforms of inositol-dependent enzyme of type 1) and ATF6 (activating transcription factor 6) [163,164,165,166]. Similarly, a mutation of protein parkin leads to impaired proteosome function and ER overload, which causes the accumulation of cytotoxic fibrils and pathological protein aggregation. Mesencephalic dopaminergic neurons are rich in parkins. The loss of mesencephalic dopaminergic neurons leads to the development of Parkinson’s disease [167]. LaVoie, M.J. et al. report that neurotransmitter dopamine covalently modifies parkin in living dopaminergic cells. This process increases parkin insolubility and inactivates its E3 ubiquitin ligase function. In the brains of individuals with sporadic Parkinson disease, scientists observed decreases in parkin solubility consistent with its functional inactivation. Using a new biochemical method, investigators detected catechol-modified parkin in the substantia nigra but not other regions of a normal human brain. These findings show a vulnerability of parkin to modification by dopamine, the principal transmitter lost in Parkinson disease, suggesting a mechanism for the progressive loss of parkin function in dopaminergic neurons during sporadic Parkinson disease [168].

The mismatch between the biosynthetic load and the functional capacity of the ER leads to overload of the ER, misfolding and accumulation of inactive or chemically aggressive proteins in the lumen of the ER [169]. In order to ensure this dynamic balance, eukaryotes have developed an “unfolded protein response” (UPR). However, if this process is disturbed during ER stress, there may develop diabetes mellitus and dysfunction of β-cells of Langerhans islets in an individual [170,171,172,173]. In particular, with diabetes mellitus, when carbohydrates enter the body, the load on the endoplasmic reticulum of β-cells increases sharply. The conditions for their apoptosis are created. At the same time, glucotoxicity and lipotoxicity can disrupt the compensation of ER stress [174,175,176]. In addition to reducing insulin secretion due to of a reduction in the β-cell population, ER stress plays an important role in the occurrence of obesity and insulin resistance in adipose, liver and muscle tissue [177].

The transcription factor ATF4 regulates the expression of genes involved in amino acid metabolism, redox homeostasis and ER stress responses. In particular, Karuppagounder, S.S. et al. found, that protection from oxidative death by adaptaquin was associated with suppression of activity of the prodeath factor ATF4 [178].

Meanwhile, Lange, P.S. et al. on cellular and animal models detected, that ATF4 as a redox-regulated, prodeath transcriptional activator in the nervous system that propagates death responses to oxidative stress in vitro and to stroke in vivo [179].

### 3.3. Osmotic Stress

This kind of stress can occur as a result of the difference between the levels of intracellular and extracellular osmolality. In this case, the cells can either swell because of the influx of water, or lose water and shrink. Osmotic stress has a large effect on cell activity. It was found that mitogen-activated protein kinases (MAPKs) have a great significance in signaling pathways (for example, WNK1-SPAK/OSR1 ASK3) in osmotic stress responses, including the regulation of intracellular levels of inorganic ions and organ mitogen-activated protein kinases (MAPKs) in osmolytes [180,181,182,183]. Osmotic stress can affect the cell nucleus biophysically. Under the influence of hypoosmotic stress, a cell nucleus can significantly increase and take a smooth rounded shape. At the same time, under the influence of hyperosmotic stress, a cell nucleus can shrink and take a twisted shape. It is supposed that osmotic stress can influence gene transcription and nucleocytoplasmic transport [184,185,186]. Mild hypoosmotic stress induces stabilization of R loops in ribosomal genes and this way it provokes the nucleolispecific DNA damage response, which is governed by the ATM- and Rad3-related (ATR) kinase. Activation of ATR in nucleoli strongly depends on Treacle, which is necessary for efficient recruitment/retention of TopBP1 in nucleoli. Subsequent ATR-mediated activation of ATM causes repression of nucleolar transcription [187,188,189,190].

The compromised MAPK signaling pathways lead to the pathological processes connected with diverse diseases including neurodegenerative disorders and cancer such as amyotrophic lateral sclerosis, Parkinson’s disease, and Alzheimer’s disease. The MAPK signaling pathways (p38 and JNK) are activated by different types of cell stress such as osmotic, genotoxic, and oxidative stress, and also by proinflammatory cytokines such as interleukin 1β and tumor necrosis factor-α. The p38 MAPK pathway is involved in the processes of neuroinflammation mediated by glial cells, in particular, microglia and astrocytes [191,192].

### 3.4. Link between Cellular Senescence, Oxidative Stress and Chronic Stress

Cellular aging loses the ability of cells to divide. Therefore, this process is also called replicative aging. In addition, with cellular aging, the efficiency of DNA repair decreases, the synthesis of proteins, RNA and ATP slows down, oxidative stress and mitochondrial dysfunction occur. Cell aging is believed to be involved in the aging mechanisms of the human body [193,194].

The authors of the article identified mutations in the mitochondrial genome associated with aging. All of them were located in the coding region of the mitochondrial genome. Most of them were localized in the genes encoding the protein subunits of the mitochondrial respiratory chain enzymes. We believe that these mutations contributed to the occurrence of oxidative stress in cells and the human body [195].

One of the mechanisms of cellular aging is the shortening of telomeres (regions on the ends of chromosomes) [196]. It happens because of the fact that the region of the chromosome, where the DNA polymerase is located before replication, is not replicated by it. Therefore, the chromosomes are shortened with each replication cycle, i.e., at each cell division. After a certain number of divisions, a significant shortening of telomeres occurs and the cell stops dividing. Telomere shortening can contribute not only to the aging of the cell and the whole organism, but also to various diseases [196,197,198,199]. In particular, Stein, J.Y., et al. found that traumatic stress which occurs in soldiers after being captured contributes to the appearing of shorter telomeres [197]. Our research group found that short telomeres are associated with coronary heart disease and type 2 diabetes mellitus [198,199]. In addition, the authors of the article found that telomeres became shorter under chronic stress caused by prolonged exposure to high air temperatures for more than 40 days in the very hot summer of 2010 [200,201].

In addition, telomere shortening and senescence associated with factors such as epigenomic damage, strong mitogen–associated signals and activation of tumor suppressors [194,202]. Smoking and unfavorable environmental factors worsen senescence and the ageing of the whole organism [194,203,204]. Moreover, these processes are often linked with chronic stress, diabetes, hypertension, coronary heart disease and other age-related diseases [194,205]. It is necessary to mention that these factors contribute to the occurrence of oxidative stress, which in its turn can cause ageing of cells and the whole organism [206,207,208,209]. Wang, L. et al. studied the mechanisms of thrombocyte ageing [206]. The given analysis was carried out using beta-galactosidase, which is associated with ageing. Meanwhile the researchers defined beta-galactosidase activity at pH 6.0 in senescent cells [208,209]. In addition, the properties of thrombocytes in the process of senescence were investigated. It turned out that the senescence of thrombocytes can be explained by the influence of oxidative stress on these cells. Meanwhile, a stimulating effect on ageing have inductors of apoptosis. A group of scientists found that the addition of resveratrol efficiently postponed thrombocytes senescence and ameliorated thrombocytes storage lesion [206,209].

An important characteristic ofmost of senescent cells is the secretory phenotype (SASP) associated with senescence. The SASP is one of the most interesting features of aging cells. With the use of SASP it is possible to explain the link of cellular senescence with aging of a human organism and diseases which are associated with age [210,211]. SASP contain proteins which can have complex effects on different types of cells. For example, the biphasic WNT modulator secreted frizzled-related protein 1 and interleukin (IL)-6 and IL-8, which can stimulate or inhibit WNT signaling cell proliferation, depend on the physiological context. Chronic WNT signaling can drive both differentiated cells and stem cells into senescence. Many SASP components directly or indirectly promote inflammation, which is particularly important in the role of cellular senescence in aging and age-related diseases. These factors include IL-8 and IL-6, a variety of monocyte chemoattractant proteins and macrophage inflammatory proteins, and proteins, regulating multiple aspects of inflammation such as granulocyte-macrophage colony-stimulating factor. As it is known, chronic inflammation may trigger or make significant contributions to many degenerative and hyperplastic age-related diseases [194,210,211]. Senescent cells can cause degenerative changes, including with the use of their secreted proteins from their cellular SASP [212,213]. Ageing cells can disrupt healthy tissue structures, causing dysfunction of tissues. SASPs of senescent astrocytes can be a reason of age-related neurodegeneration. In particular, these SASPs can be triggers of Parkinson’, Alzheimer’s diseases and brain disorders [194,214,215]. Moreover, SASPs of senescent cells ofsmooth musculature, epithelium and endothelium may be included into mechanisms of the occurrence and development of age-related cardiovascular disease [194,216]. Therefore, senescent cells may be a trigger on the occurrence and development of many age-related pathologies, such as atherosclerosis, metabolic syndrome, type 2 diabetes mellitus, hypertension, cardiovascular diseases, insulin insensitivity, macular degeneration, emphysema, and chronic obstructive pulmonary disease.

## 4. The Relationship of Chronic Stress with the Occurrence and Development of Atherosclerosis

In addition to neurodegenerative diseases, the chronic stress can lead to the occurrence and development of atherosclerosis [14,15,19,20,21,217]. Some research groups have shown that atherosclerosis often develops in people who experience steady stress. In particular, Heidt, T. et al. detected that chronic stress induced monocytosis and neutrophilia in the human organism [218]. While investigating the source of leukocytosis in mice, the scientists found that stress activates upstream hematopoietic stem cells. During chronic stress in mice, sympathetic nerve fibers released surplus noradrenaline, which signaled bone marrow niche cells to decrease CXCL12 levels through the β3-adrenergic receptor. This suggests that hematopoietic stem cell proliferation was elevated. There was an increased output of neutrophils and inflammatory monocytes. When atherosclerosis-prone Apoe (−/−) mice were exposed to chronic stress, accelerated hematopoiesis promoted plaque features linked with vulnerable lesions which cause stroke and myocardial infarction in humans [218].

It is necessary to note that acute myocardial infarction (AMI) and stroke are risk factors for atherosclerosis. A trigger of these pathologies can be chronic stress. In particular, Arnold, S.V. et al. compared patients with AMI with high and low level of chronic stress. They considered chronic stress for the period of 30 days before AMI happened and during one year after AMI. They also considered AMI during two years after AMI happened. It was found that patients with high level of chronic stress died twice more often than patients with low level of chronic stress [219]. Zhao, Z. et al. investigated on animal model the effect of chronic stress on the brain after stroke. It was detected that chronic stress has a strong negative impact on key components of cells, participating in the remodelling of vessels of nervous system. Besides, chronic stress worsens vascular disorders [220] Takotsubo syndrome occupies a special place among these pathologies. Takotsubo syndrome is acute cardiac disease with a clinical presentation that of an acute coronary syndrome. Chronic stress often occurs before Takotsubo syndrome. During the acute stage of the syndrome, in patients may develop such complications as arrhythmias and heart failure including cardiogenic shock, cardiac arrest and rupture. Scally, C. et al. found that Takotsubo syndrome has such characteristics as changes in the distribution of monocyte subsets, an increase in systemic proinflammatory cytokines and a myocardial macrophage inflammatory infiltrate [221]

A number of scientists have identified a relationship between endoplasmic reticulum stress and the occurrence of atherosclerosis [222,223,224,225,226,227]. It was found that ER stress occurs in cells involved in the occurrence and development of atherosclerosis, including endothelial cells and macrophages. ER stress also affects the processes of atherosclerosis diseases by coordinating protein and lipid metabolism, inflammatory response and cell apoptosis. According to recent data, molecular signaling pathways between ER stress and the NLRP3 (nucleotide-binding oligomerization domain-like receptor family, pyrin domain-containing 3) inflammasome play a large role in atherogenesis [140,228,229,230]. ER stress is thought to affect NLRP3 inflammasome activation through reactive oxygen species generation, the unfolded protein response, metabolic products of calcium or lipids [231].

Cellular stress may be one of the causes of atherosclerosis occurrence. Tabas, I. assumes that cellular stress contributes to the formation of vulnerable plaques, including the secretion of pro-inflammatory, procoagulant, and proteolytic molecules by macrophages and also the death of intimal smooth muscles cells, macrophages, and possibly endothelial cells. A keyfactor in plaque vulnerability is the necrotic core in particular, because macrophage debris promotes inflammation, plaque instability, and thrombosis. Plaque necrosis arises from a combination of lesional macrophage apoptosis and defective clearance of these dead cells. This process is named efferocytosis [232].

Bonomini, F. et al. supposes that the reactive nitrogen (RNS)and reactive oxygen (ROS) species are the important endogenous sources produced by non-enzymatic and enzymatic (nicotinamide adenine dinucleotide phosphate oxidase (NADH oxidase), myeloperoxidase (MPO), and lipoxygenase (LO)) reactions that may be balanced with anti-oxidative compounds (vitamins, polyphenols and glutathione (GSH)) and enzymes (peroxiredoxins (Prdx), glutathione peroxidase (Gpx), paraoxonase (PON), and superoxide dismutase (SOD)). However, the oxidative and anti-oxidative imbalance causes the involvement of cellular proliferation and migration signaling pathways and macrophage polarization leads to the formation of atherogenic plaques [233,234].

The researchers of our laboratory discovered the association of mitochondrial genome mutations with atherosclerosis (m.652delG, m.3256C>T, m.13513G>A, m.14846G>A, m.1555A>G, m.12315G>A, m.14459G>A, m.5178C>A, m.652insG, m.3336T>C and m.15059G>A). This study was carried out using a new original quantitative method developed by the authors of the article on the basis of pyrosequencing technology [18,235,236,237,238,239]. Threshold values of the heteroplasmy level of 11 mitochondrial genome mutations were detected, after which in patients atherosclerosis began to occur or the protective effect of these mutations began to appear (Table 2 and Table 3) [18,235,236,237,238,239].

The authors of the article created two cybrid cultures, one of which contained an atherogenic mutation of the mitochondrial genome (m.12315G>A), and the other contained an antiatherogenic mutation mtDNA (m.1555G>A) [240,241,242,243,244]. These cultures was created on the basis of a monocytic origin culture of THP-1. Platelet donors for the creation of cybrid cultures were study participants who had a heteroplasmic level of interest mutations above the threshold level. Then, the oxygen respiration level was measured in these cybrid cultures. In a cybrid culture containing an atherogenic mutation, the level of oxygen consumption was lower than in a native culture of THP-1. At the same time, in cybrid cells containing an antiatherogenic mutation, the level of oxygen consumption was higher than in the native THP-1 culture. We assume that this data may indicate a negative effect of oxidative stress on a cybrid culture containing an atherogenic mutation of the mitochondrial genome [240,241,242,243,244].

In addition, our research team found that the number of copies of mtDNA in a cybrid culture containing atherogenic mutation m.12315G>A was significantly lower than in a native culture of THP-1. We assume that this fact indicates the occurrence of replicative stress in this cybrid culture [240,241,242,243,244].

Most of mutations, linked with atherosclerosis, cardiovascular diseases and aging, belong to genes of subunits of mitochondrial respiratory chain enzymes (*MT-ND1*, *MT-ND2*, *MT-ND5*, *MT-ND6*, *MT-CYTB*) [18,235,245,246,247,248,249]. The occurrence of such mutations can lead to energy deficiency in cells, for the reason of enzyme dysfunction [18,235,245,246,247,248]. This, in its turn, can lead to mitochondrial dysfunction and oxidative stress in cells and in the human organism. Such processes can cause the occurrence and development of diseases, and also speed up the aging process [207,210]. Part of the detected mutations belongs to tRNA genes (*MT-TL1*, *MT-TL2*) and rRNA (MT-*RNR1*). Mutations in these genes can lead to dysfunction of tRNA and rRNA [18,235,245,246,247]. As a result, the amount of normal RNA and proteins in mitochondria decreases. In mitochondria, metabolism is disturbed. The cell begins suffer from shortage of ATP molecules, which ultimately leads to oxidative stress.

## 5. Conclusions

**Figure 2:**
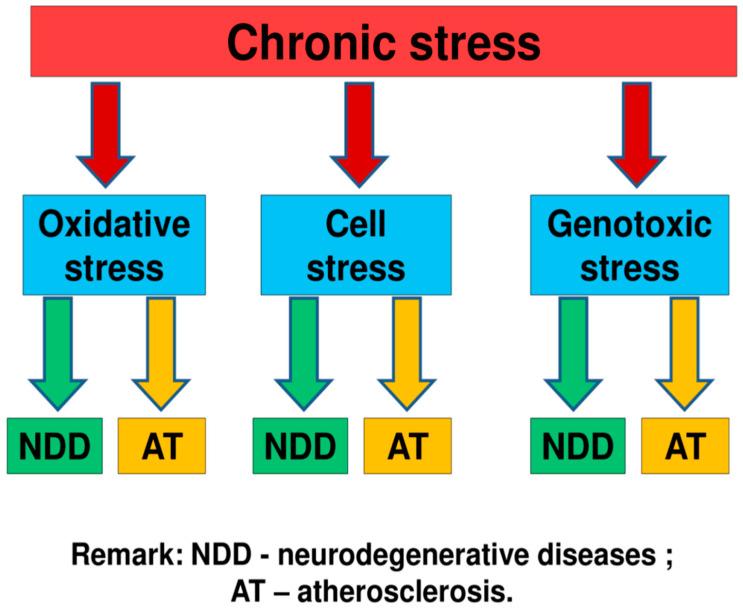
Some mechanisms of the link of chronic stress with neurodegenerative diseases and atherosclerosis [8,9,10,11,12,13,14,15,16]. We suppose that the most important role in this process play molecular and cellular types of stress. Among molecular types of stress, the largest contribution to pathogenesis of neurodegenerative diseases and atherosclerosis can make oxidative and genotoxictypes of stress.

An analysis of literary sources allowed us to conclude that, under the influence of chronic stress, the metabolism in the human body can be disrupted, mutations of the mitochondrial and nuclear genome, dysfunction of cells, and their compartments can occur. As a result of these processes, molecular and cellular types of stress can occur. Therefore, chronic stress can be one of the causes of the occurrence and development of neurodegenerative diseases and atherosclerosis [8,9,10,11,12,13,14,15,16]. We suppose that among molecular types of stress, the largest contribution to pathogenesis of neurodegenerative diseases and atherosclerosis can make oxidative and genotoxictypes of stress (Figure 2) [8,9,10,11,12,13,14,15,16].

The relevance of the present review article is proven by the fact that there are few articles, in which general mechanisms of occurrence and development of chronic stress, neurodegenerative diseases and atherosclerosis were considered. However, recently, experimental articles have been published on this theme [8,9,10,11,12,13,14,15,16]. In our article we have made an attempt to give a theoretical ground for these researches. We hope that this article will be useful to scientists and doctors involved in research in this area.

## Figures and Tables

**Table 1:** Reactive oxygen species [30,31,32,33,34].

Number	Reactive Oxygen Species	Chemical Formula
1	Superoxide anion	O_2_^−^
2	Hydroxyl radical	OH-
3	Nitrogen oxide anion	NO-
4	Hydrogen peroxide	H_2_O_2_
5	Peroxynitrite anion	ONOO^−^
6	Hydroperoxide radical	HO_2_-
7	Singlet oxygen	O^1^_2_
8	Hypochlorite anion	OCl^−^
9	Hypobromite anion	OBr^−^
10	Hypoiodic anion	OI^−^
11	Alkyldioxyl radical	ROO-
12	Alkoxyl radical	RO-
13	Organic radical	R-

**Table 2:** Value of heteroplasmy level of mtDNA mutations in atherosclerotic plaques [18,235,236,237,238,239].

Genes	Mutations	Threshold Value of Heteroplasmy Level (%)
*MT-TL2*	m.12315G>A	7.6
*MT-RNR1*	m.652delG	20.4
*MT-ND1*	m.3336T>C	6.6
*MT-TL1*	m.3256C>T	15.4
*MT-CYTB*	m.14846G>A *	17.6
*MT-RNR1*	m.652insG	20.1
*MT-RNR1*	m.1555A>G *	17.5
*MT-ND6*	m.14459G>A	4.5
*MT-ND2*	m.5178C>A	6.5
*MT-ND5*	m.13513G>A *	32.4
*MT-CYTB*	m.15059G>A	24.6

*—Antiaherogenic mutations.

**Table 3:** The threshold value of heteroplasmy level of mtDNA mutations in thickened intima-medial layer of carotid arteries [18,235,236,237,238,239].

Genes	Mutations	Threshold Value of Heteroplasmy Level (%)
*MT-TL2*	m.12315G>A	10.4
*MT-RNR1*	m.652delG	21.6
*MT-ND1*	m.3336T>C	7.5
*MT-TL1*	m.3256C>T	16.4
*MT-CYTB*	m.14846G>A *	17.4
*MT-RNR1*	m.652insG	20.2
*MT-RNR1*	m.1555A>G *	19.6
*MT-ND6*	m.14459G>A	4.6
*MT-ND2*	m.5178C>A	6.4
*MT-ND5*	m.13513G>A *	33.6
*MT-CYTB*	m.15059G>A	26.4

*—Antiaherogenic mutations.

## Data Availability

Not applicable.
